# Establishment of chicken dedifferentiated fat (DFAT) cells as a preadipocyte cell line for adipogenic differentiation studies

**DOI:** 10.1016/j.psj.2025.106299

**Published:** 2025-12-23

**Authors:** Reiko Hagiwara, Yoshinao Oki, Takashi Nishi, Koichiro Kano

**Affiliations:** a Laboratory of Cell and Tissue Biology, College of Bioresource Sciences, Nihon University, 1866 Kameino, Fujisawa, Kanagawa 252-0880, Japan; b Oncology Innovation Center, Fujita Health University, 1-98 Dengakugakubo, Kutsukake-cho, Toyoake, Aichi 470-1192, Japan

**Keywords:** Ceiling culture, Chicken preadipocyte cell line, Dedifferentiation, Differentiation, Mature adipocyte

## Abstract

Avian adipocyte differentiation differs from mammalian adipocyte differentiation; however, no useful preadipocyte cell line has been established to study avian adipocyte differentiation. This study investigated whether dedifferentiated fat (DFAT) cells derived from chick mature adipocytes have the same characteristics as stromal vascular fraction (SVF) cells derived from adipose tissues and whether DFAT cells can be used as an avian preadipocyte cell line. The floating top layer, which contained mature adipocytes, was isolated from chick abdominal fat tissue by collagenase digestion and filtration. To induce spontaneous dedifferentiation, the isolated mature adipocytes were cultured using the ceiling culture method. After 14 days of culture, the proliferation and differentiation potential of the resulting DFAT cells was evaluated. The isolated mature adipocytes successfully dedifferentiated into DFAT cells and actively proliferated in ceiling culture. The proliferative capacity of DFAT cells was similar to that of the SVF cells, although the ability of DFAT cells to differentiate into mature adipocytes was significantly higher than that of SVF cells. Subcultured DFAT cells maintained normal proliferation and differentiation into adipocytes for at least 33 passages. This study demonstrated that chicken DFAT cells provide a readily established preadipocyte cell line derived from mature adipocytes, which can be used as a model for investigating adipogenic differentiation in chickens.

## Introduction

Chickens (*Gallus gallus domesticus*) are among the key livestock as well as an important model animal for biological research. Despite the fact that it has been previously reported that adipocyte differentiation and adipogenesis in chickens vary compared to mammals (Matsubara et al., 2005; Farmer, 2006, 2008; W. Wang, et al., 2017), the exact details of the molecular mechanisms involved in adipocyte differentiation and adipogenesis in chickens remain unknown.

Research on chicken adipocyte differentiation has used chick embryonic fibroblasts (CEFs) disaggregated from 5- to 12-day-old embryos (Hassan, et al., 2014; Kim, et al., 2020, 2024). These CEFs are highly proliferative and, upon adipocyte differentiation, express specific genes and develop into cells with multiple lipid droplets, making them suitable for studies of adipocyte differentiation. However, CEFs represent a heterogeneous population derived from tissues at various developmental stages and possess characteristics distinct from those of preadipocytes in adipose tissue (Smas and Sul, 1995; Wolins, et al., 2006).

Various studies have focused on differentiating chicken adipocytes using the stromal vascular fraction (SVF) isolated from embryonic and post-hatch chicken adipose tissue. This approach may better reflect the in vivo context and provide a stable in vitro primary culture system for chick adipogenic differentiation (Van, 1985). However, SVF is a heterogeneous population that includes not only preadipocytes but also several other cell types present in adipose tissue, such as erythrocytes, macrophages, monocytes, endothelial cells, fibroblasts, and pericytes. All of these cell types can influence adipocyte differentiation (Ramsay, et al., 1992). Furthermore, adipose tissue contains various cell types capable of differentiating into fat cells (Zuk, et al., 2002), including multipotent stem cells, adipoblasts committed to the adipocyte lineage, preadipocytes expressing early- or late-adipocyte differentiation markers, and immature adipocytes that have not yet accumulated lipid droplets. Based on the above, a primary culture system based on this fraction is inappropriate for assessing the molecular basis of adipocyte differentiation in chickens. In order to examine thus the molecular basis of adipogenic differentiation and adipogenesis in chickens, establishing a useful preadipocyte cell line is essential.

Two cell lines have been established to investigate chick adipogenic differentiation as follows; DF-1 cell line was spontaneously immortalised using embryonic fibroblasts derived from 10-day chick embryos (Himly, et al., 1998; Lee, et al., 2021). Immortalized chicken preadipocytes (ICP) cell line was based on fibroblasts immortalised by exogenous genes into SVF cells isolated from the abdominal adipose tissue of 10-day-old chickens (Wang, et al., 2017). Nevertheless, embryonic-derived fibroblasts and adipose tissue-derived SVF cells consist of various cell types. These cell lines are not thus homogeneous cell populations derived from preadipocytes. Embryonic fibroblast-derived cell lines often present characteristics different from those of tissue preadipocytes as they are generated from embryos before the formation of white adipose tissue (Smas and Sul, 1995; Wolins, et al., 2006). DF-1 cell line derived from chick embryonic fibroblasts is also hypothesised presenting characteristics that vary from those of tissue preadipocytes. In ICP cell lines foreign genes have been introduced using retroviral vectors for immortalisation, although the introduction of exogenous genes has generated various concerns, including the risk of insertional mutagenesis, residual expression, genetic alterations and the disruption of intercellular gene networks (Hawley, 2008; Hu, 2014; Lee, et al., 2018). With respect to the abovementioned concerns, the chicken preadipocyte cell lines, DF-1 and ICP cell lines are not necessarily suitable for assessing the molecular mechanisms of adipocyte differentiation.

When mouse, rat, rabbit, feline, porcine, bovine and human adipocytes are cultured using the ceiling culture method, mature adipocytes spontaneously dedifferentiate to generate dedifferentiated fat (DFAT) cells (Yagi, et al., 2004; Matsumoto, et al., 2008; Nobusue, et al., 2008; Ohta, et al., 2008; Nobusue and Kano, 2010; Kikuta, et al., 2013; Kono, et al., 2014; Oki, et al., 2022). DFAT cells contain a homogeneous cell population as they are derived from mature adipocytes isolated from adipose tissue. DFAT cells present a fibroblast-like cell morphology and they actively proliferate. It has been previously reported that when DFAT cells were triggered towards adipogenic differentiation by employing an adipogenic cocktail, expression of early differentiation marker genes, such as CCAAT/enhancer-binding protein (*C/EBP*) family members and peroxisome proliferator-activated receptor gamma 2 (*PPARγ2*) were increased, followed by the corresponding expression of adipocyte-specific genes and redifferentiation into mature adipocytes with an accumulation of lipid droplets in the cytoplasm. These properties were preserved in each subculture even after long-term culturing and passaging. In addition, DFAT cells that were transplanted subcutaneously could redifferentiate into mature adipocytes *in vivo* and develop highly angiogenic fat pads that morphologically resemble normal subcutaneous adipose tissue (Yagi, et al., 2004; Nobusue, et al., 2008). Based on these results, it is considered that chicken DFAT cells that present the same proliferative and differentiative properties as those of the chicken preadipocytes, can be utilised to generate chicken mature adipocytes.

The present study focused on establishing a preadipocyte cell line resulting from chick mature adipocytes using the ceiling culture method. We demonstrated that isolated chick mature adipocytes could dedifferentiate into fibroblast-like cells and then significantly proliferate in the ceiling culturing method. In addition, it was demonstrated that chicken DFAT (c-DFAT) cells are able to proliferate and differentiate into mature adipocytes. It is thus suggested that c-DFAT cells provide a useful cell model for investigating adipogenic differentiation in chickens.

## Materials and methods

### Animals and diet

Male chicks (*Gallus gallus domesticus*, White Leghorn strain) were purchased from a commercial hatchery immediately after hatching. Chicks were housed in thermostatically controlled battery cages (25°*C* ± 3°C) with a 12 h light/dark cycle. Chicks were fed with a commercial diet during the early growth phase (21 % crude protein, 4 % crude fat and 3,200 kcal/diet metabolisable energy) and water was provided *ad libitum*. Two-week-old chicks were utilised in all the experiments. Animal handling and care protocols were approved by Kobe University Animal Experiments Committee and were performed based on the guidelines for the Act on Welfare and Management of Animals (Japanese Governmental Law No. 105).

### Isolation of chick mature adipocytes

Sterile dissection was conducted in order to excise abdominal adipose tissue following an ethical procedure for rapid decapitation. A quantity lower than 1 g of adipose tissue was extensively washed using phosphate-buffered saline (PBS) supplemented with 80 µg/ml kanamycin (Sigma-Aldrich, St Louis, MO), was cut into small pieces and cultured in Dulbecco's modified Eagle's medium (DMEM, pH 7.4; Nissui Pharmaceutical, Hiroshima, Japan) containing 0.1 % collagenase (Type II, Sigma-Aldrich) and 2 % bovine serum albumin (BSA; Fraction V, Sigma-Aldrich) supplemented with 25 mM 2-[4-(2-Hydroxyethyl)-1-piperazinyl] ethanesulfonic acid, 1.8 mg/ml NaHCO_3_ and 80 µg/ml kanamycin. Samples were gently shaken at 37°C for 30 min to digest adipose tissues and the cell suspension was then filtered using a 150 and 100 µm nylon mesh (PP-150 N; Kyoshin Rikoh, Tokyo, Japan) to remove undigested tissue debris. The filtered cells were extensively washed using centrifugation at 135 × *g* three times for 3 min. Primary adipocytes floating in the top layer and SVF cells at the bottom of the tube were independently collected and purified by centrifugation three times.

### Microscopic analysis

The floating primary adipocytes were fixed in 4 % formaldehyde (Wako, Osaka, Japan) for 10 min, washed with PBS, and stained with 1 μg/ml 4′, 6-diamino-2-phenylindole (DAPI; Sigma-Aldrich) in PBS for 30 min. The number of mature adipocyte nuclei was assessed by counting 1,029 cells in a hemocytometer under a fluorescence microscope (Olympus, Tokyo, Japan). Data from three trials were averaged.

### Cell culture

Isolated mature adipocytes (approximately 1 × 10^5^ cells/12.5 cm^2^) were positioned in culture flasks (BD Falcon, Glendale, AZ) with DMEM supplemented with 10 % fetal bovine serum (FBS; Bulimba, Australia). Since the flasks were filled with medium that created an air-free environment for the cells, they were then inverted and incubated at 37°C in a humidified atmosphere containing 5 % CO_2_ and 95 % air. The cells were floating in the medium and adhered to the top inner surface (ceiling) of the flask. Approximately 1 week later, cells had attached steadily in the flask’s ceiling and modified into fibroblast-like shapes with no visible fat droplets. The medium was replenished every 4 days until 100 % confluency was achieved. SVF cells were resuspended in DMEM containing 10 % FBS and 80 µg/ml kanamycin in a new test tube and were seeded in 35 mm primary culture dishes at a density of 9.6 × 10^4^ cells/dish, in which they were cultured at 37°C in a humidified atmosphere containing 5 % CO_2_ and 95 % air until 100 % confluency.

### Evaluation of proliferative activity

Cell proliferation activity was evaluated by measuring cell numbers over time. Cells were cultured for 8-12 days after seeding. Following the culture period, cells were washed three times with PBS, then harvested using trypsin treatment and centrifugation. Cell numbers were subsequently measured with a hemocytometer and expressed as cells per square centimeter. In each experiment, cell numbers were determined using five technical replicates.

### Induction into adipocyte differentiation

SVF and c-DFAT cells were triggered towards adipocyte differentiation based on a previous report (Matsubara, et al., 2008). Confluent cells were washed thoroughly using serum-free DMEM to eliminate the effect of serum on adipocyte differentiation. Cells were cultured in serum-free DMEM containing 10 µg/ml transferrin (Wako, Osaka, Japan), 10 µg/ml insulin (Wako) and 0.1 % BSA supplemented with or without 10 % fatty acid (FA) concentration liquid (FACL) containing a various long-chain FAs (GIBCO, Grand Island NY, USA) for 12 days. The medium was changed every 3 days until the cells were ready for experimentation.

### Lipid staining

Modifications in cell morphology and accumulation of cytoplasmic lipid droplets were detected and images were acquired under a phase-contrast microscope (Olympus, Tokyo, Japan). In order to obtain histochemical data, lipid droplets were visualised using Oil Red O (ORO) staining. Cells were washed using PBS and fixed with 4 % formalin–PBS solution for 1 h at room temperature. Following fixation, cells were washed once with distilled water and stained with filtered 0.5 % ORO (Sigma-Aldrich), that was dissolved using isopropyl alcohol for 15 min at room temperature. Cells were then washed with distilled water and culture dishes with ORO-stained cells were visualised with macroscopic observation.

### Real-time reverse transcription–polymerase chain reaction analysis

Before FA treatment and every four days since then, total RNA was extracted from cells using TRIzol reagent (Invitrogen, Carlsbad, CA) based on the manufacturer’s instructions. In order to detect the mRNA expression of delta-like homolog 1(*DLK1*), peroxisome proliferator-activated receptor gamma (*PPARγ*), CCAAT/enhancer-binding protein alpha (*C/EBPα*) and adipocyte FA-binding protein (*aP2*), real-time reverse transcription (RT)–polymerase chain reaction (PCR) analysis was conducted using the Applied Biosystems 7300 Real-Time PCR System (Applied Biosystems). The oligonucleotide sequences of the PCR primers are presented in Table 1. Total RNA (1 µg) digested with DNase I were subjected to RT with the use of random primers and moloney murine leukaemia virus reverse transcriptase (Invitrogen). Each RT-reaction served as a template in a 50 µl PCR reaction containing 0.5 mM of each primer and SYBR green master mix (Bio Whittaker Molecular Applications). The temperature cycles used were as follows: 94°C for 3 min followed by 35 cycles at 94°C for 30 s, 63°C for 1 min and 72°C for 1 min. SYBR green fluorescence was detected at the end of each cycle to detect the PCR product levels that were formed during the cycle. In order to correct for the differences in the template DNA levels, the expression data were presented in a ratio over 18S ribosomal RNA as an internal control (primer sequence prese ted in Table 1). Each sample was performed in triplicate.

**Table 1:** Primer sequence for real-time RT-PCR.

Accession number	Gene name	Primer sequence: 5′ to 3′
EU288039	*DLK1*	F: TGTGTGCCCAGGGATTTACAGGA
		R: ACCTGCACCAATATCTGTGCACG
AF163811	*PPARγ*	F: TACATAAAGTCCTTCCCGCTGACC
		R: TCCAGTGCGTTGAACTTCACAGC
X66844	*C/EBPα*	F: GTGCTTCATGGAGCAAGCCAA
		R: TGTCGATGGAGTGCTCGTTCT
AF432507	*aP2*	F: GAGTTTGATGAGACCACAGCAGA
		R: ATAACAGTCTCTTTGCCATCCCA
AF173612	*18S ribosomal RNA*	F: TAGATAACCTCGAGCCGATCGCA
		R: GACTTGCCCTCCAATGGATCCTC

### Glycerol-3-phosphate dehydrogenase activity

Glycerol-3-phosphate dehydrogenase (GPDH) activity of EC 1.1.1.8 was used as a late marker of adipocyte differentiation. Cell extracts for GPDH activity were conducted with some modifications made to the approach previously described by Wise and Green (Wise and Green, 1979). In brief, after removing the medium, cells were washed twice with iced cold PBS. Cells were recovered by scraping with a rubber policeman in 25 mM Tris (pH 7.5; Sigma-Aldrich) supplemented with 1 mM ethylenediaminetetraacetic acid (EDTA; Nacalai, Kyoto, Japan) and sonicated for 10 s at 150 W (oscillator regular output). After centrifugation at 12,800 × *g* for 5 min at 4°C, the supernatant fraction was further centrifuged at 100,000 × *g* for 1 h at 4°C. The obtained supernatant fraction was used in a subsequent assay. GPDH activity was determined following a method described by Kozak and Jensen (Kozak and Jensen, 1974). The standard mixture contained 5 mM dihydroxyacetone phosphate (Sigma-Aldrich), 0.5 mM reduced nicotinamide adenine dinucleotide (Oriental Kobo, Tokyo, Japan) and 50 mM tri-ethanol amine (Wako) supplemented with 10 mM EDTA and 10 mM β-mercaptoethanol. An absorbance modification at 340 nm was measured using a spectrophotometer (UV-1200; Shimadzu, Kyoto, Japan). One enzyme activity unit was defined as the amount necessary for oxidisation of one nmol of NADH/min. The cytosolic protein concentrations of the supernatants were determined following the method of Lowry et al. (Lowry, et al., 1951).

### Statistical analysis

Data on the number of nuclei per isolated mature adipocyte were analysed using the Chi-square test with Yates' correction for continuity to determine statistical differences between experiments. The mean cell number, mean GPDH activity, and comparisons among gene expression data were analysed for significant differences nonparametrically using Mann–Whitney's U test with StatView software (version 4.5, Abacas Concepts Inc.). A probability of *P* < 0.05 and 0.01 was considered significant in all statistical analyses.

## Results

### Morphological changes of mature adipocytes into c-DFAT cells by the ceiling culture method

**Fig. 1:**
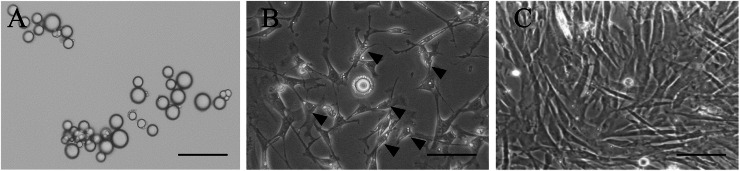
Morphological changes in dedifferentiating cells from chick mature adipocytes via the ceiling culture method. (A) Morphology of isolated mature adipocytes. Following 2 days of the ceiling culturing method, adipocytes were observed to contain a large single lipid droplet. (B) Following 4 days of the ceiling culturing methods, few adipocytes were observed to contain many small lipid droplets around the large lipid droplet. The majority of the other adipocytes converted into fibroblast-like cells and actively proliferated but retained small lipid droplets in the cytoplasm (arrowhead). (C) Following 8 days of the ceiling culturing approach, all cells were converted to fibroblasts without lipid droplets in the cytoplasm and reached confluence. Bars, 100 µm.

To obtain uniform single-cell suspensions of mature adipocytes and in order to prevent contamination by preadipocytes, fibroblasts and/or SVC cells, adipose tissue was digested in a collagenase solution under gentle agitation (Nobusue, et al., 2008; Nobusue and Kano, 2010). The dissociated adipocytes were purified by multiple rounds of centrifugation and filtration that resulted in adipocytes with a large, single lipid droplet. In a preliminary experiment, we confirmed that mature chicken adipocytes could be obtained at about 0.4 - 1 × 10^6^ cells per 1 g of chicken abdominal adipose tissue (data not shown). To test whether the isolated mature adipocytes were mononucleate cells without tightly attached SVF cells, the nuclei of isolated mature adipocytes were stained with DAPI, and the number of mononuclear mature adipocytes was counted using a fluorescence microscope. Table 2 shows that 99.6 % of the isolated cells were mononuclear, mature adipocytes, indicating that the cells were a highly homogeneous fraction. Following 2 days in the ceiling culture, mature adipocytes in culture flasks filled with medium floated to the top and adhered to the top inner surface of the flask (Fig. 1A). Following 4 days of ceiling culture, a few adipocytes with several small lipid droplets formed across the large lipid droplets. The majority of the other cells demonstrated fibroblast-like morphology and actively proliferated but maintained small lipid droplets in the cytoplasm (Fig. 1B). Small lipid droplets in the cytoplasm then disappeared following every day of culturing. Following 8 days of culturing, cells transformed to fibroblasts without lipid droplets in the cytoplasm reaching to 100 % confluency (Fig. 1C).

**Table 2:** The number of DAPI-stained nuclei in adipocytes isolated from adipose tissue.

Total adipocytes	No. of cells with 1 nucleus/cell	No. of cells with 2 or more nuclei/cell
377	376	1
320	319	1
332	330	2
1029	1,025 (99.6 %)^A^	4 (0.4 %)^B^

Note: Values represent the total number of cells counted in three independent experiments. Values in parentheses indicate the percentage of three trials. Means within a row lacking a common uppercase superscript differ significantly (*P* < 0.01).

### Proliferative properties of c-DFAT cells generated from mature adipocytes

**Fig. 2:**
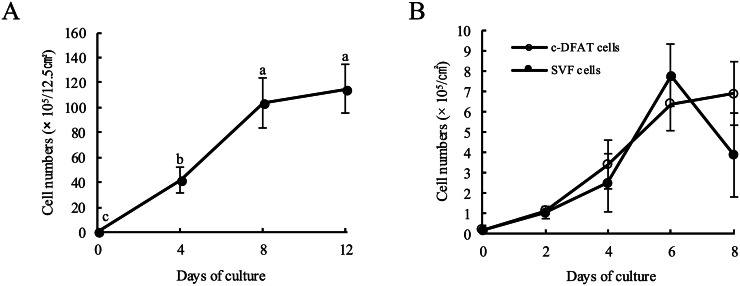
Proliferative properties of c-DFAT cells. (A) The proliferative potential of c-DFAT cells generated by ceiling culture of chicken mature adipocytes was determined. The c-DFAT cells were collected by trypsinisation and centrifugation following at least 4 days of culturing with the ceiling culture approach and the number of cells per flask was determined. Bars indicate the mean ± *SD* of five independent experiments performed on at least a triplicate of flasks. a, b, c: Letters above the bars indicate statistical significance; different letters indicate significant differences between the number of culturing days (*P* < 0.05). (B) Proliferation potential of c-DFAT and SVF cells. SVF and c-DFAT cells were inoculated in culture dishes at an initial density of 10^3^ cells/cm^2^ and cultured in DMEM supplemented with 10 % FBS. Cells were then harvested every 2 days from the start of the culture using trypsinization and centrifugation, and the cell count per dish was subsequently determined.

The number of c-DFAT cells generated from approximately 10^5^ cells/flask (12.5 cm^2^) of mature adipocytes isolated from chicken adipose tissue of <1 g by the ceiling culture method was then investigated (Fig. 2A). Ceiling culture of mature adipocytes at approximately 10^5^ cells/flask resulted in a rapid growth of c-DFAT cells generated from mature adipocytes to 1 × 10^7^ cells/flask following 8 days of culturing and then became confluent. These findings suggest that x100 times more c-DFAT cells can be collected by ceiling culture of mature adipocytes, that is significantly effective.

The growth potential of c-DFAT cells was presumed to be low as these cells originated from mature adipocytes in the G_0_ phase. The *in vitro* proliferative capacity of c-DFAT cells was thus evaluated and compared with that of SVF cells (Fig. 2B). Both c-DFAT cells and SVF cells promptly increased up to 6 days following culturing. The c-DFAT cells then reached 100 % confluency and proliferation stopped. In SVF cells, the number of cells decreased following 8 days of culturing, as some of the cells detached from the culture dish. These results demonstrated that c-DFAT cells presented a growth ability that was suitable for *in vitro* experiments.

### Characteristics of adipocyte differentiation in c-DFAT cells

An investigation was then focused on whether adipogenic stimulation could trigger c-DFAT cell differentiation into functional adipocytes. Based on the above, c-DFAT cells were thus cultured using an adipogenic medium containing 10 % FACL mainly consisting of various types of FAs.

**Fig. 3:**
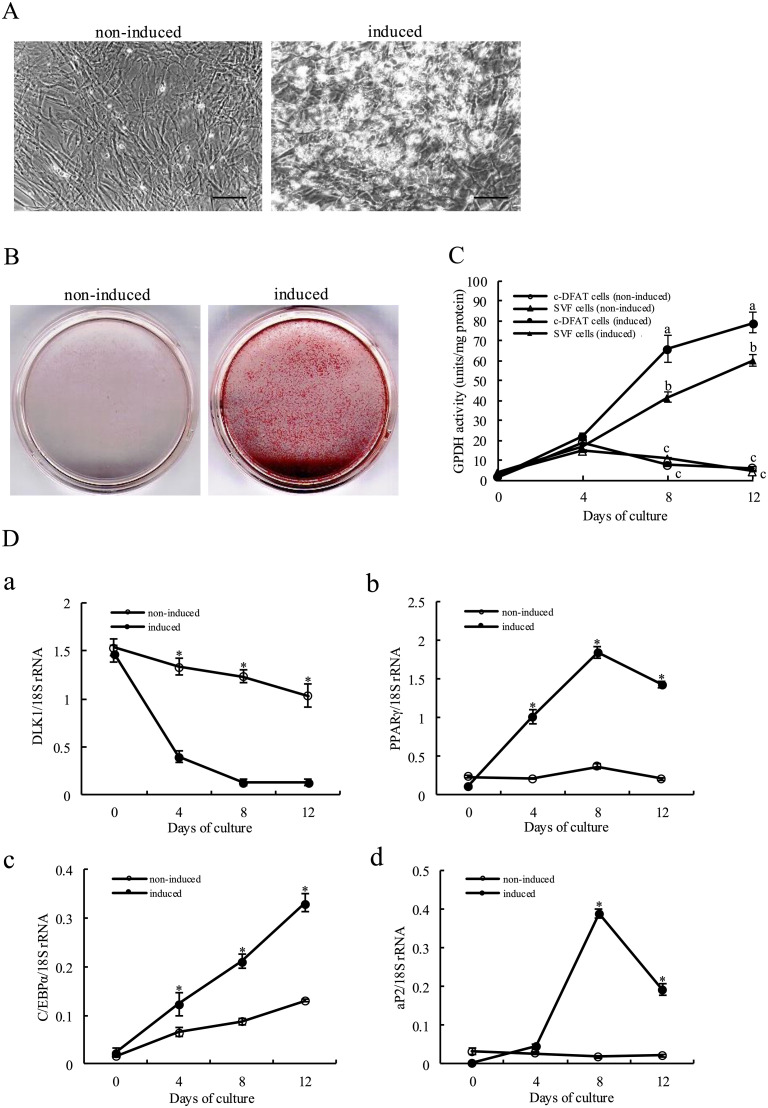
Confluent cells were maintained for 12 days in serum-free DMEM supplemented with 10 µg/ml transferrin (TF), 10 µg/ml insulin (INS), and 0.1 % bovine serum albumin (BSA) with or without 10 % fatty acid concentration liquid (FACL) treatment. (A) Microscopic observation. Modifications in cell morphology and cytoplasmic lipid droplets in the cells were observed and images were acquired using a phase-contrast microscopy on day 12 following the induction of adipocyte differentiation. Left: Non-induced cells; Right: induced cells. Bars, 100 µm. (B) ORO staining. For histochemical analysis, cells were fixed with formalin and stained using ORO solution. Images of the culture dishes of ORO-stained cells were acquired macroscopically 12 days following adipogenic induction. Left: Non-induced cell; Right: induced cell. (C) Glycerol-3-phosphate dehydrogenase (GPDH) activity. The GPDH activity of c-DFAT cells with (●) or without (○) adipogenic differentiation stimulation and that of SVF cells with (▲) or without (△) adipogenic differentiation were determined every 4 days during the culture period. Values are means ± *SD* of six independent experiments performed on at least a triplicate of dishes. a, b, c: Letters above the bars indicate statistical significance; different letters indicate significant differences same day of culture day (*P* < 0.05). (D) The mRNA expression levels of *DLK1* (a), *PPARγ* (b), *C/EBPα* (c) and *aP2* (d) were determined using real-time RT-PCR. Data are presented as a ratio with the expression level of 18S rRNA, that was used as an internal control. Values are presented as the mean ± *SD* of triplicate samples. *Significant differences between the control and induced cells within the culturing day (*P* < 0.05).

**Fig. 4:**
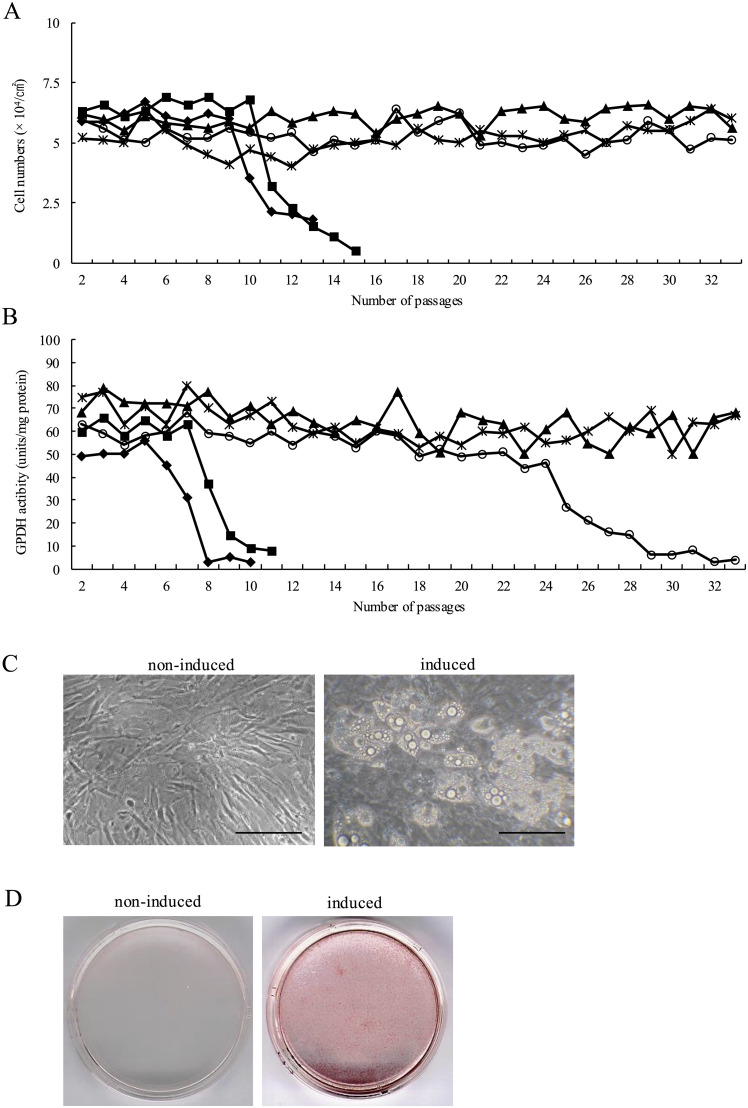
The effect of long-term subculture on the proliferation and differentiation potential of c-DFAT cells. (A) The proliferative potential of c-DFAT cells in each subculture was evaluated. The c-DFAT cells were obtained from five individual chicks, were inoculated in culture dishes at an initial density of 10^3^ cells/cm^2^ and were cultured in DMEM supplemented with 10 % FBS. On the 10th day, cells were harvested by trypsinisation and the number of cells per dish was determined. (B) GPDH activity of c-DFAT cells in each subculture. The confluent c-DFAT cells established from five individual chicks were cultured in serum-free DMEM supplemented with 10 µg/ml TF, 10 µg/ml INS, 0.1 % BSA and 10 % FACL. GPDH activity was determined on the 10th day following adipogenic induction. ▲, Clone 1; *, clone 2; ○, clone 3; ■, clone 4; ◆, clone 5. (C) Microscopic observation of c-DFAT cells in the 30th generation. Modifications in cell morphology and cytoplasmic lipid droplets in the cells were observed images were acquired under phase-contrast microscopy on day 12 following the induction of adipocyte differentiation. Left: Non-induced cells; Right: induced cells. Bars, 100 µm. (D) ORO staining in c-DFAT cells at 30th generation. For histochemical data, cells were fixed with formalin and stained with ORO solution. Culture dishes of ORO-stained cells and images were acquired macroscopically on the 12th day following adipogenic induction. Left: Non-induced cell; Right: induced cell.

The c-DFAT cells that did not induce adipogenic differentiation maintained a fibroblast-like appearance even after 12 days of culture and did not contain lipid droplets in the cytoplasm (Fig. 3A). In contrast, when c-DFAT cells were induced to differentiate into adipocytes, their morphology changed to a polygonal shape 4 days after adipogenic induction, with small lipid droplets accumulating in the cytoplasm. By 12 days post-induction, the number of cells containing large lipid droplets in the cytoplasm had increased (Figs. 3A and 4C). Fig. 3B presents ORO staining following 12 days of adipogenic induction. The c-DFAT cells triggering adipogenic differentiation demonstrated a significant increase in lipid droplets compared to non-induced cells. These results suggest that c-DFAT cells differentiated into mature adipocytes by stimulating adipogenic differentiation.

In c-DFAT cells and SVF cells that were utilised as controls with or without adipogenic stimulation, the GPDH activity was then measured, that is normally utilised as a late marker of adipocyte differentiation. As presented in Fig. 3C, the GPDH activity of c-DFAT and SVF cells rapidly increased up to 12 days following the stimulation of adipogenesis. GPDH activity of c-DFAT cells at day 8 and 12 following adipogenesis stimulation was significantly higher compared to that of the SVF cells. c-DFAT and SVF cells without adipogenic stimulation demonstrated a slight increase in GPDH activity for the first 4 days following the differentiation stimulation. Nevertheless, the activity thereafter decreased to less than 10 U/mg of protein for the remaining culture period.

Fig. 3D presents the mRNA expression pattern of preadipocyte marker gene and adipocyte-specific genes during adipocyte differentiation using real-time RT-PCR. *DLK1*, a preadipocyte marker gene, showed high expression during differentiation induction but rapidly decreased by day 4, resulting in a significant difference. Subsequently, *DLK1* gene expression remained low throughout the culture period. In the non-induced control group, *DLK1* gene expression remained consistently high throughout the entire culture period (Fig. 3Da). The expression levels of *PPARγ* gene, a key regulator of adipocyte differentiation, rapidly increased up to eight days following adipogenesis induction. In contrast, the *PPARγ* gene expression in control cells was not modified during the culturing period (Fig. 3Db). Importantly, the expression levels of the *C/EBPα* gene rapidly increased up to 12 days following adipogenesis stimulation while *C/EBPα* gene expression levels were significantly higher compared to those of the control cells throughout the culturing period (Fig. 3Dc). Expression levels of the *aP2* gene, that transports FAs, was not modified until day 4 following adipogenesis stimulation. Subsequently, *aP2* gene expression levels rapidly increased up to day 8 following adipogenesis stimulation while they declined 12 days after. In control cells, *aP2* gene expression levels were not modified during the culturing period (Fig. 3Dd). These results indicate that c-DFAT cells express adipocyte-specific genes during adipocyte differentiation.

### Effect of long-term subculturing on the proliferation and differentiation of c-DFAT cells

Cell lines are commonly characterised by the maintenance of high proliferative ability and stable differentiation potential during subculturing, while they are valuable for their consistent and reproducible culture strategy. In order to test whether c-DFAT cells could be utilised as a cell line, c-DFAT cells established from five individual chickens were assessed with respect to their proliferation and differentiation in each subculture (Fig. 4). c-DFAT cells isolated from five individual chicks were cultured in DMEM supplemented with 10 % FBS and the cell number per dish was determined following 10 days of culturing. The number of c-DFAT cells isolated from three of the five chicks was maintained for 33 passages, while the number of c-DFAT cells derived from the remaining two chicks decreased rapidly in passages 9 and 10 (Fig. 4A). In order to evaluate whether c-DFAT cells present an adipogenic differentiation potential in each subculture, GPDH activity was assessed 12 days following adipogenic stimulation (Fig. 4B). Nevertheless, GPDH activity of the two clones decreased rapidly at passages 6 and 7, while one clone did not differ until 24 passages but then decreased until passage 33. Cell morphology of c-DFAT cells and the accumulation of fat droplets in each subculture following adipocyte differentiation triggering was further assessed. As demonstrated in Fig. 4C and D, fat droplets accumulated in the cytoplasm and ORO-stained adipocytes were monitored all over the culture dish 12 days following differentiation stimulation, even in c-DFAT cells that were cultured for 30 passages. These results did not present any difference among passages. These results suggest that subcultured c-DFAT cells maintained normal proliferation and differentiation into adipocytes for at least 33 passages in 2 of 5 (40 %) individuals. The c-DFAT cells thus present the optimal characteristics for a cell line.

## Discussion

The present study presented the establishment of novel chicken preadipocyte cell lines, called c-DFAT cells and are derived from chick abdominal adipose tissue. The c-DFAT cells originated from a homogeneous cell population that derived from a single fraction of mature adipocytes. These cells proliferated significantly until they reached confluency and differentiated into mature adipocytes more effectively compared to SVF cells originating from chick adipose tissue. The growth rate of c-DFAT cells was stable with increasing passage number and c-DFAT cells derived from two of the five chicks were successfully maintained and differentiated up to at least 33 passages. It is thus suggested that c-DFAT cells and the strategy described herein provide a novel model system for evaluating the mechanism of chicken adipocyte differentiation.

In order to study the molecular mechanism of adipocyte differentiation, obtaining a pure preadipocyte fraction is essential. Nevertheless, studies of adipocyte differentiation in mammals and birds, including humans, have been previously performed using SVF cells, except for a few small experimental animals that depended on useful immortalised preadipocyte cell lines (Ramsay and Rosebrough, 2003; Shang, et al., 2014). This culturing system comprises various stromal cells and vascular cells and elimination of their impact is challenging. In order to resolve this crucial issue, it is convenient to isolate preadipocytes by cloning SVF, despite the fact that this is challenging for the two following main reasons. Because no suitable molecular markers have been identified for flow cytometric sorting of preadipocytes, isolating them from the SVF is not feasible. Using the limiting dilution approach is challenging, as the number of preadipocytes in SVF is limited (Yoshimura, et al., 2006; Traktuev, et al., 2008) and their proliferative potential is reduced when subcultured. As mature adipocytes contain large lipid droplets in the cytoplasm, they float up to the upper fraction following collagenase treatment. Significantly, over 99 % of the upper fraction obtained from chicken adipose tissue can be isolated as a single fraction of mature adipocytes (Table 2). Furthermore, this finding is consistent with results previously reported in various mammalian species, including humans (Matsumoto, et al., 2008; Nobusue, et al., 2008; Nobusue and Kano, 2010). In the present study, 0.4–1 × 10^6^ mature adipocytes could be collected from approximately 1 g of chicken adipose tissue following collagenase digestion and filtration (data not shown). When approximately 10^5^ cells/flask (12.5 cm^2^) of mature adipocytes were cultured for 8 days using the ceiling culture approach, c-DFAT cells were produced for approximately 10^7^ cells/flask (Fig. 2A). Moreover, c-DFAT cells number was increased approximately six times following 8 days of performing the subculture and the proliferative potential was maintained even after repeated subcultures (Figs. 2B and 3A). These results suggest that c-DFAT cells can be utilised to effectively produce large volume of pure cells from a small quantity of adipose tissue.

Our group previously reported that SVF cells isolated from chicken abdominal adipose tissue can differentiate into mature adipocytes after stimulation in a serum-free medium supplemented with FA (Matsubara, et al., 2008). In the present study, it was examined whether c-DFAT cells can differentiate into adipocytes in a process similar to that of SVF cells under the culturing conditions previously described. Following stimulation of adipogenic differentiation, c-DFAT cells were modified from a fibroblast-like appearance to a polygonal one and lipid droplets were concentrated in the cytoplasm (Fig. 3A). ORO staining of positive cells increased following stimulation of adipogenic differentiation and was observed throughout the culture dish (Fig. 3B). GPDH activity in c-DFAT cells rapidly increased following adipogenic stimulation (Fig. 3C). These results were in accordance with studies that utilised adipogenic differentiation of SVF cells derived from chicken abdominal adipose tissue. Nevertheless, GPDH activity of the c-DFAT cell group was significantly higher compared to that of the SVF cell group following differentiation stimulation and the cell number concentrating lipid droplets in the cytoplasm was also higher compared to those of the SVF cell group. These results suggest that the differentiation potential of c-DFAT cells is higher than that of SVF cells. Despite the fact that DFAT cells are produced from a single fraction of mature adipocytes (Matsumoto, et al., 2008; Nobusue, et al., 2008; Nobusue and Kano, 2010), SVF comprises several cells, including preadipocytes (Butterwith, et al., 1992; Zuk, et al., 2002). Consequently, c-DFAT cells appear to present a higher rate of differentiation into adipocytes compared to SVF cells.

In mammals, terminally differentiated cells cannot reverse the differentiation process in most of the cases (Andrés and Walsh, 1996; Walsh and Perlman, 1997). Mature adipocytes present a specific morphology with a single large droplet in the cytoplasm and their proliferation is ceased due to the G_0_ phase of the cell cycle (MacDougald and Lane, 1995; Tang, et al., 2003; Rosen and MacDougald, 2006). In the present study, we attempted to produce c-DFAT cells by stimulating spontaneous dedifferentiation of chick mature adipocytes using the ceiling culture method. Accordingly, the mature adipocytes were gradually modified to a fibroblast-like morphology and actively proliferated while decreasing the number of cytoplasmic fat droplets (Fig. 1B). The cells were then transformed to fibroblasts with no fat droplets in their cytoplasm (Fig. 1C). These fibroblasts exhibited high levels of *DLK1*, a preadipocyte marker, which rapidly decreased by day 8 of adipocyte differentiation induction. Subsequently, *DLK1* expression remained significantly low until day 12 of culture (Fig. 3Da). In contrast, *DLK1* gene expression, without induction of adipogenic differentiation, remained significantly elevated until day 12 of culture. The expression of adipocyte-specific genes, such as *PPARγ, C/EBPα*, and *aP2*, in these cells was markedly lower than that observed after differentiation induction (Fig. 3Db-d). Additionally, the expression of adipocyte-specific genes in the control group, which did not undergo induction of adipocyte differentiation, remained low throughout the culture period (Fig. 3Db-d). These results suggest that mature chicken adipocytes spontaneously dedifferentiate to the preadipocyte stage to generate c-DFAT cells, aligning with findings in rodents, humans, cats, pigs, and cattle (Yagi, et al., 2004; Matsumoto, et al., 2008; Nobusue, et al., 2008; Ohta, et al., 2008; Nobusue and Kano, 2010; Kikuta, et al., 2013; Kono, et al., 2014; Oki, et al., 2022). Based on these results, we propose that a homogeneous preadipocyte population can be obtained from mature chicken adipocytes using the ceiling culture approach, without the need for limiting dilution cloning.

One of the vital objectives of this study was to examine whether c-DFAT cells derived from mature chicken adipocytes could be utilised as preadipocytes for examining the differentiation mechanism of chicken adipocytes. It was thus assessed whether c-DFAT cells differentiate into mature adipocytes through a systematic approach of adipocyte differentiation following stimulation of adipogenic differentiation (Gregoire, et al., 1998). It is established that during adipogenesis, members of the *C/EBP* family and *PPARγ* are implicated in terminal differentiation in the adipogenic process by subsequent transactivation of adipocyte-specific genes (Gregoire, et al., 1998; Rangwala and Lazar, 2000; Matsubara, et al., 2005; Wang, et al., 2008, 2012). When c-DFAT cells were stimulated towards differentiation into adipocytes, *PPARγ* and *C/EBPα* gene expression patterns were significantly increased compared to those of controls (no induction) in the early stages of adipogenesis (Fig. 3Db and c). The *aP2* is a member of the fatty acid-binding protein family and plays a key role in intracellular FA transport and metabolism, particularly in maintaining glucose and lipid homeostasis (Elmasri, et al., 2009). It was initially identified as an adipocyte-specific protein that is predominantly expressed in mature adipocytes. Consequently, *aP2* is identified as a middle and late marker of adipocyte differentiation *in vitro* (Spiegelman, et al., 1983). The expression level of *aP2* mRNA in c-DFAT cells increased rapidly 4–8 days following stimulation of adipogenic differentiation (Fig. 3Dd). In addition, GPDH activity implicated in triglyceride production, was also rapidly increased from four to eight days following stimulation of differentiation (Fig. 3C). The c-DFAT cells differentiated at the same time into mature adipocytes in which lipid droplets were concentrated in the cytoplasm (Fig. 3A and C). This result suggests that mature adipocyte-derived c-DFAT cells are dedifferentiated cells until the preadipocyte phase and can differentiate into mature adipocytes following adipogenic stimulation. It was thus concluded that c-DFAT cells might represent a valuable model for *in vitro* studies on the mechanism of adipogenic differentiation.

Primary cultured cells from living tissue have a limited lifespan; each time these cells divide, the telomeric DNA in the ends of their chromosomes shortens (Hayflick and Moorhead, 1961; Harley, et al., 1990). Shortening of the telomeric DNA through repeated passaging cultures marks the beginning of cellular senescence (Blasco, 2007), while cell arrest proliferation is characterised as senescent because telomeric DNA is lost and its essential sequence is eventually deleted. In contrast, several cell lines established from tumours have active telomerase that synthesises shortened telomeric repeats (Kim, et al., 1994). Their proliferative ability is stably maintained over time by maintaining telomere length. Many cell lines derived from normal somatic cells have thus been established by transfecting the telomerase reverse transcriptase (*TERT*) gene. In the present study, c-DFAT cells originating from two of the five chicks retained their proliferation potential and differentiate into mature adipocytes for at least 33 passages (Fig. 4). The aetiology for long-term maintenance of the proliferative potential of c-DFAT cells generated from mature adipocytes of the two individuals has not been yet established. Our group has previously reported that mouse, pig and bovine DFAT cells generated by spontaneous dedifferentiation of mature adipocytes, retain their proliferative potential and their ability in redifferentiating into adipocytes (Nobusue, et al., 2008; Nobusue and Kano, 2010; Oki, et al., 2022). Telomerase expression in each proliferation of normal somatic cells, including mature adipocytes, is suppressed and shortened (Harley, et al., 1990; Bouazzaoui, et al., 2013). *TERT* levels in rat and human DFAT cells are in contrast 2 to 2.5 times higher compared to mesenchymal stem cells (MSCs) originating from adipose tissue and decrease senescence compared to MSCs (Gao, et al., 2012; Watson, et al., 2014). In addition, stimulation of reprogramming in somatic cells results in telomere elongation and fibroblasts isolated from telomerase-deficient mice inhibit stimulation of reprogramming (Marion, et al., 2009). Based on these results, it was presumed that the reprogramming that occurs during spontaneous dedifferentiation of mature adipocytes triggers telomere expression. Based on the above, it is considered that c-DFAT cells from two individuals cultured effectively for long-term passaging in the present study released telomerase expression during dedifferentiation and retained high *TERT* levels, while DFAT cells from other individuals were suppressed or lost during repeated subculturing. The mechanism of initialisation and telomerase expression during dedifferentiation of mature adipocytes requires further elucidation. Moreover, the effect of passaging on the telomerase activity of DFAT cells should be also evaluated.

In addition, c-DFAT cells are generated by stimulation of spontaneous dedifferentiation of mature adipocytes. The c-DFAT cells can be subcultured for an extended period without immortalisation. Consideration of the effects of the introduction of foreign genes is not essential as c-DFAT cells can be simply and efficiently isolated from mature adipocytes that can be isolated from only 1 g of adipose tissue. These results indicate that c-DFAT cells can be generated from mature adipocytes, irrespective to the species, individual, age, sex and adipose tissue depot site. The benefit of the approach for generation of DFAT cells has also been demonstrated in DFAT cells generated from mature adipocytes of mice (Yagi, et al., 2004; Nobusue, et al., 2008), rats (Ohta, et al., 2008), rabbit (Kikuta, et al., 2013), felines (Kono, et al., 2014), pigs (Nobusue and Kano, 2010), bovine (Oki, et al., 2022) and humans (Matsumoto, et al., 2008).

In summary c-DFAT cells are a unique preadipocyte cell line that encompasses a homogeneous cell population derived from a single fraction of chicken mature adipocytes while they possess of the majority of preadipocytes properties and can preserve their ability to proliferate and differentiate into mature adipocytes in all subcultures, even after 33 passages. Thus, it is concluded that c-DFAT cells are preadipocytes derived from dedifferentiated mature chicken adipocytes and can be a valuable cell model for investigating the mechanism of adipogenic differentiation studies.

## Funding

This work was supported by grants from the Ministry of Education, Culture, Sports, Science and Technology of Japan (grant no. 19K06444 to K. Kano).

## CRediT authorship contribution statement

**Reiko Hagiwara:** Writing – original draft, Investigation, Formal analysis, Data curation. **Yoshinao Oki:** Writing – original draft, Investigation, Formal analysis, Data curation. **Takashi Nishi:** Investigation, Data curation. **Koichiro Kano:** Writing – review & editing, Supervision, Resources, Project administration, Methodology, Investigation, Funding acquisition, Formal analysis, Data curation, Conceptualization.

## Disclosures

The authors declare that they have no known competing financial interests or personal relationships that could have appeared to influence the work reported in this paper.
